# Maternal Body Mass Index and Recommended Gestational Weight Gain in a Middle Eastern Setting

**DOI:** 10.1007/s10995-023-03816-z

**Published:** 2023-11-13

**Authors:** Tawa Olukade, Husam Salama, Sawsan Al-Obaidly, Mai AlQubaisi, Hilal Al-Rifai

**Affiliations:** 1 Department of Pediatrics, Hamad Medical Corporation, Doha, Qatar; 2 Neonatal Intensive Care Unit, Hamad Medical Corporation, Doha, Qatar; 3 Department of Obstetrics and Gynecology, Hamad Medical Corporation, Doha, Qatar

**Keywords:** Gestational weight gain, Body mass index, Qatar, Middle east

## Abstract

**Objective:**

Maternal body mass index (BMI) and gestational weight gain (GWG) are modifiable risk factors that influence pregnancy outcomes. We examined the association between the two factors in pregnant women in Qatar with regard to the GWG recommendations by the Institute of Medicine (IOM) in 2009.

**Methods:**

We performed a population-based retrospective cohort analysis of 3547 singleton births, using routinely collected data from a Middle Eastern hospital database.

**Results:**

The mean maternal age was 29.7 ± 5.5 years, prepregnancy BMI was 27.5 ± 5.8 kg/m^2^, GWG was 9.58 kg ± 6.87 kg and gestational age at birth was 38.5 ± 1.9 weeks. In line with IOM recommendations, we found that higher BMI was correlated with decreased GWG and BMI was significantly associated with GWG even after adjusting for maternal age, parity, and infants’ gestational age at birth. Nonetheless, GWG in more than one-third of women who were overweight or obese exceeded the IOM recommendation.

**Supplementary Information:**

The online version contains supplementary material available at 10.1007/s10995-023-03816-z.

## Significance

Gestational weight gain is an important factor that influences short- and long-term pregnancy outcomes in women and their infants. The present study findings show that while many women achieved optimum weight gain during pregnancy, a significant proportion of who are overweight or obese, exceeded recommended weight gain.

**Supplementary Information:**

The online version contains supplementary material available at 10.1007/s10995-023-03816-z.

## Objectives

Excessive gestational weight gain (GWG) has been linked to short- and long-term adverse fetal and maternal outcomes. It is a modifiable risk factor associated with an increase in rates of cesarean deliveries, infants large for gestational age, postpartum weight retention in women, and the development of early childhood obesity (Hernandez, 2012; Li et al., 2018, 2019; Ludwig et al., 2013; Siega-Riz et al., 2009; Sridhar et al., 2014, 2016; Voerman et al., 2019). GWG recommendations and assessment are often based on a woman’s prepregnancy body mass index (BMI). Obesity is currently a global health problem among men and women; with more than 1.9 billion people reported to be overweight, and 650 million living with obesity (WHO, 2017). With this global trend, increasing numbers of women are likely to begin pregnancy classified with either overweight or obesity.

Qatar is a high-income country with a large number of people having overweight or living with obesity. According to the 2006 Qatar World Health Survey, which included Qatari citizens and residents, 39% of the population was classified as overweight, 28.8% as obese, and 3.3% as morbidly obese (National Health Authority (Qatar), 2006). Of the Qatari citizens alone, 75% were classified as overweight or obese, and more than 66% of expatriates as either overweight or obese. Moreover, according to the 2012 STEPwise survey of only Qatari citizens, the mean BMI in the Qatari population aged 18 to 64 years were 28.8 kg/m^2^ in men and 29.5 kg/m^2^ in women (Haj Bakri and Al-Thani, 2013). In that survey, 70.1% of participants had a BMI of ≥ 25 kg/m^2^, the average BMI of nonpregnant women aged 18–44 years was 28.4 kg/m^2^, and of those women, 25.4% were classfied as overweight and 36.4% as having obesity. These proportions were similar in a Qatar Biobank annual report (Qatar Biobank, 2017). Obesity, metabolic syndromes, and lifestyle-related disorders are nationally prioritized issues in Qatar, and efforts have been ongoing to mitigate these health burdens. Although pregnant women receive dietary and lifestyle education, not all may receive adequate education about BMI and expected GWG unless it is particularly indicated. In several studies, conducted in different parts of the world such as the United States, Sweden, Asian nations, and other countries, GWG decreased with a higher BMI, and a significant proportion of women gained more weight than recommended (Arora & Tamber Aeri, 2019; Johansson et al., 2016; Cheney et al., 2017; Power et al., 2018). The Institute of Medicine (IOM) recommend the following GWG for various groups according to BMI: 12.5–18.0 kg (underweight), 11.5–16.0 kg (normal weight), 7.0–11.5 kg (overweight), and 5.0–9.0 (obese).

We did not find reports of any large study on GWG in Qatar, therefore we examined the pattern of GWG among pregnant women in Qatar. Evidence generated could inform policy and practice in the characterization and prediction of GWG in Qatar. The objective of this study was to examine the association between prepregnancy BMI and GWG among women with singleton pregnancies with regard to the 2009 recommendations of the IOM (Rasmussen & Yaktine, 2009).

## Methods

### Study Design

We performed a retrospective analysis of data retrieved from the population-based Perinatal Neonatal Outcomes Research Study in the Arabian Gulf (PEARL)–Peristat Registry in Qatar. This observational research registry was funded by the Qatar National Research Fund and sponsored by the Medical Research Centre of Hamad Medical Corporation. The primary aim of the registry was to study fetal and maternal outcomes in the perinatal–neonatal period in Qatar, and it contained routinely collected data extracted from hospital records for women and newborns. The research complied with all the relevant regulations and institutional policies, was conducted in accordance with the tenets of the Helsinki Declaration, and was approved by the Medical Research Centre, Hamad Medical Corporation. The requirement for consent was waived because of the retrospective nature of the study.

### Setting and Participants

Data retrieved from the registry for this analysis included data from public hospitals, which account for approximately 86% of all deliveries in Qatar (Haj Bakri and Al-Thani, 2013). In addition, health care quality in private hospitals is comparable with that in public hospitals.

Data collection for the registry started in January 2017 in the largest maternity hospital (Women’s Wellness Research Centre) and in March 2017 in three other hospitals, Al-Wakra, Al-Khor, and Cuban hospitals. Information on participants with completed data entry from January to June 2017 was retrieved for this analysis. All 9249 participants who gave birth to singletons at 24 weeks of gestation and above were eligible. The inclusion criterion was data for GWG; no exclusion criteria were used. Data from 3547 (37.6%) of the women were available for computing GWG and were included in the analysis. According to Johansson and colleagues, data for GWG is often lacking in many birth registers; such data were available for only one third of women in the Swedish Medical Birth Register (Johansson et al., 2016). In evaluating our sample for selection bias, we observed that the proportions of demographic variables did not differ significantly between the women with and without GWG data; differences ranged from 0.4 to 2.8%, although the proportion of women with diabetes during pregnancy was 11% higher among the women with GWG data than among those without. Our sample was therefore representative of the population of women who delivered singleton infants within the study period.

### Primary Outcome Variable

GWG (in kilograms) was defined as the difference between measured weight at delivery and measured or self-reported prepregnancy weight (International Association of Diabetes in Pregnancy Study Group (IADPSG) Working Group on Outcome Definitions et al., 2015). Maternal prepregnancy weight and weights at each antenatal care visit were routinely documented in the electronic medical records of every pregnant woman. Prepregnancy weight in this study was defined as documented weight before confirmation of pregnancy or weight at early antenatal booking (< 13 weeks’ gestation). According to a review by the Working Group on Outcome Definitions of the International Association of Diabetes in Pregnancy Study Group (IADPSG), whose aim is to establish consensus definitions for maternal and fetal outcomes, GWG had various definitions in the literature. The definition finally adopted by the working group was “Weight from preconception (preferable) (measured or self-reported) or within 3 months of conception, or if not available, at first pregnancy visit within first trimester, until the last measured weight during pregnancy (within 4 weeks of delivery)” (International Association of Diabetes in Pregnancy Study Group (IADPSG) Working Group on Outcome Definitions et al., 2015). Data for weight at delivery were available for approximately 99.5% of the study population, but prepregnancy weights and weights at early antenatal booking (< 13 weeks’ gestation) were available for only 21% (1939/9249) and 24.8% (2298/9249) of the sample, respectively. To conform with the IADPSG definition, we used the booking weight at < 13 weeks’ gestation as the prepregnancy weight for cases in which the prepregnancy weight was unavailable (Online Resource 1). The same approach was used in computing BMI.

### Secondary Outcome Variables

The proportions of women who gained less than, within, or more than the GWGs recommended by the IOM for each BMI category served as the secondary outcome variables.

### BMI and Other Covariables

Prepregnancy BMI (in kilograms per square meter) was calculated as the ratio of prepregnancy weight (in kilograms) divided by height (in square meters). It was examined as both a continuous variable and a categorical variable, and BMI categories were defined as underweight (≤ 18.5 kg/m^2^), normal weight (18.5–24.9 kg/m^2^), overweight (25.0–29.9 kg/m^2^), and obese (≥ 30.0 kg/m^2^) (WHO, 2006).

#### Women

Maternal age at delivery was assessed in groups: ≤19, 20–24, 25–29, 30–34, and ≥ 35 years. For parity, women were considered nulliparous or uniparous/multiparous. Nationality was classified as Qatari or non-Qatari. The presence of diabetes (overt or gestational) was coded as “yes” (1) or “no” (0).

#### Newborns

Sex was classified as male or female. Gestational age at delivery was assessed in groups: ≤28, 29–32, 33–36, and ≥ 37 weeks.

### Statistical Analysis

Descriptive statistics were calculated as numbers and percentages or as means and standard deviations (SDs) as applicable to show the distribution of variables. Both GWG and BMI had normal distributions. First, we estimated the mean GWG for each covariable and examined the association of each covariable with GWG by using Pearson’s correlation coefficient. Then we performed univariable and multivariable linear regression to examine and test the association between GWG and prepregnancy BMI while adjusting for the effects of significant covariables. We utilized two models for linear regression: BMI was a continuous covariable in the first model and a categorical covariable in the second model. A univariable general linear model, UNIANOVA (univariable analysis of variance) was used to estimate each association of BMI category with GWG. The IBM-SPSS UNIANOVA command provides results for regression analysis and analysis of variance for a dependent variable based on one or more factors. Finally, the mean GWG in each BMI category was compared to the IOM’s recommended ranges. Furthermore, the proportions of women who gained less than, within, and more than the IOM’s recommendation were also estimated in terms of the 25th, 50th, and 75th percentiles. To perform statistical analysis, we used IBM-SPSS 22 statistical software (IBM Corporation, Armonk, NY, USA). Statistical significance was determined in 95% confidence intervals (CI) and indicated by *p* levels of < 0.05.

## Results

Table 1 lists the demographic characteristics and the mean GWG of the 3547 women in this study. The mean maternal age was 29.7 ± 5.5 years, prepregnancy BMI was 27.5 ± 5.8 kg/m^2^ and around three-fifth of these women; n = 2194 (61.9%) were aged 25–34 years. Majority of the women who gave birth were non-Qatari with term pregnancies (mean gestational age at birth, 38.5 ± 1.9 weeks) who had at least one previous parous experience. Using the WHO BMI cut-off mentioned earlier, 95 (2.7%) were classified as underweight before pregnancy, 1247 (35.2%) as normal weight, 1143 (32.2%) as overweight, and 1061 (29.9%) as living with obesity before pregnancy. Prepregnancy BMI data was missing for one woman.

**Table 1:** Participant characteristics and gestational weight gain pattern in 3547 singleton births

Variable	n (%)	Mean gestational weight gain, kilograms (SD)	Pearson *r*	*p* value*
Maternal age (years)	3547			
Mean,SD	29.7 ± 5.5			
≤ 19 years	64 (1.8)	10.97 (7.09)	−0.162	< 0.001
20–24 years	575 (16.2)	11.22 (6.86)		
25–29 years	1134 (32)	10.11 (6.92)		
30–34 years	1060 (29.9)	9.34 (6.29)		
≥ 35 years	714 (20.1)	7.68 (7.14)		
Maternal nationality	3547			
Qatari	773 (21.8)	9.46 (7.3)	0.010	0.557
Non-Qatari	2774 (78.2)	9.62 (6.74)		
Parity	3547			
Nulliparous	1030 (29)	11.69 (7.09)	−0.196	< 0.001
Parity ≥ 1	2517 (71)	8.72 (6.59)		
Gestational age at delivery	3547			
Mean,SD	38.5 ± 1.9			
≤ 28 weeks	23 (0.6)	4.55 (4.82)	0.124	< 0.001
29–32 weeks	36 (1)	4.78 (4.87)		
33–36 weeks	236 (6.7)	7.49 (5.67)		
≥ 37 weeks	3252 (91.7)	9.83 (6.92)		
Prepregnancy BMI Mean,SD	3547	9.58 (6.87)	−0.285	< 0.001
BMI groups^a^	3546^b^			
Underweight	95 (2.7)	13.17 (8.27)	−0.260	< 0.001
Normal	1247 (35.2)	11.27 (6.15)		
Overweight	1143 (32.2)	9.70 (6.55)		
Obese	1061 (29.9)	7.10 (6.85)		
Diabetes in pregnancy	3547			
None	2366 (66.7)	10.34 (6.58)	−0.156	< 0.001
GDM/DM	1181(33.3)	8.07 (7.17)		
Sex of newborn	3544^b^			
Male	1783 (50.3)	9.62 (6.98)	−0.005	0.747
Female	1761 (49.7)	9.55 (6.76)		

*BMI* body mass index, *DM* diabetes mellitus, *GDM* gestational diabetes mellitus, *SD* standard deviation

^a^Institute of Medicine recommended gestational weight gain for BMI groups: 12.5–18.0 kg (underweight); 11.5–16.0 kg (normal weight); 7.0–11.5 kg (overweight); and 5.0–9.0 kg (obese)

^b^Missing data for prepregnancy BMI (1) and for the sex of newborns (3)

*Significant at *p* < 0.01

The overall mean GWG was 9.58 kg (SD = 6.87 kg), with finding showing an inverse correlation between GWG andd BMI. GWG was highest in the underweight group (mean = 13.17 kg, SD = 8.27 kg) and lowest among the women living with obesity (mean = 7.10 kg, SD = 6.85 kg; Table 1). The mean GWG decreased with increased maternal age, parity, and prepregnancy BMI. Mean GWG was also decreased in women with any form of diabetes in pregnancy. In contrast, GWG increased with gestational age of the fetus. The mean GWG with regard to the nationality of the women and sex of the newborns were equivalent.

In the univariable analysis (Table 1), we found a significant correlation between GWG and prepregnancy BMI (r[3544] = − 0.260; p = < 0.001), maternal age (r[3545] = − 0.162; p = < 0.001), parity (r[3545] = − 0.196; p = < 0.001), the gestational age of the newborn (r[3545] = 0.124; p = < 0.001), and the presence of diabetes during pregnancy (r[3545] = − 0.156; p = < 0.001). We found no correlation between nationality, gender of newborn, and GWG.

In both regression models examined; with BMI categorized or continuous,, BMI was significantly associated with GWG (Table 2). The association remained significant even after we adjusted for maternal age, parity, and the gestational age of the newborn (adjusted beta = − 0.26 kg; 95% CI − 0.29 kg, − 0.22 kg; *p* < 0.05). The adjusted regression model showed that for every unit increase in BMI (with an intercept of 12.1), GWG decreased by 0.26 units; where GWG = (− 0.26 × BMI) + 12.1. For all BMI groups, and in comparison with the women living with obesity, GWG increased by a factor of 2.11 kg in women grouped as overweight, 3.14 kg in the normal weight group, and 4.74 kg in the underweight group (Table 2).

**Table 2:** Univariable and multivariable regression models between prepregnancy BMI and gestational weight gain

BMI^a^	Unadjusted beta (95% CI)	*R* ^2^	Adjusted beta (95% CI)	*R* ^2^
Underweight	6.07 (4.69, 7.45)		4.75 (3.37, 6.12)	
Normal weight	4.17 (3.63, 4.70)	0.069	3.14 (2.59, 3.69)	0.115
Overweight	2.59 (2.05, 3.15)		2.11 (1.57, 2.65)	
Obese	Reference		Reference	
BMI as a continuous variable	−0.33 (− 0.37, − 0.29)	0.081	−0.26 (− 0.29, − 0.22)	0.121

*BMI* body mass index, *CI* confidence interval

^a^Adjusted for maternal age, parity, diabetes (gestational or nongestational), and gestational age of newborn

### GWG and IOM Recommendations

**Fig. 1:**
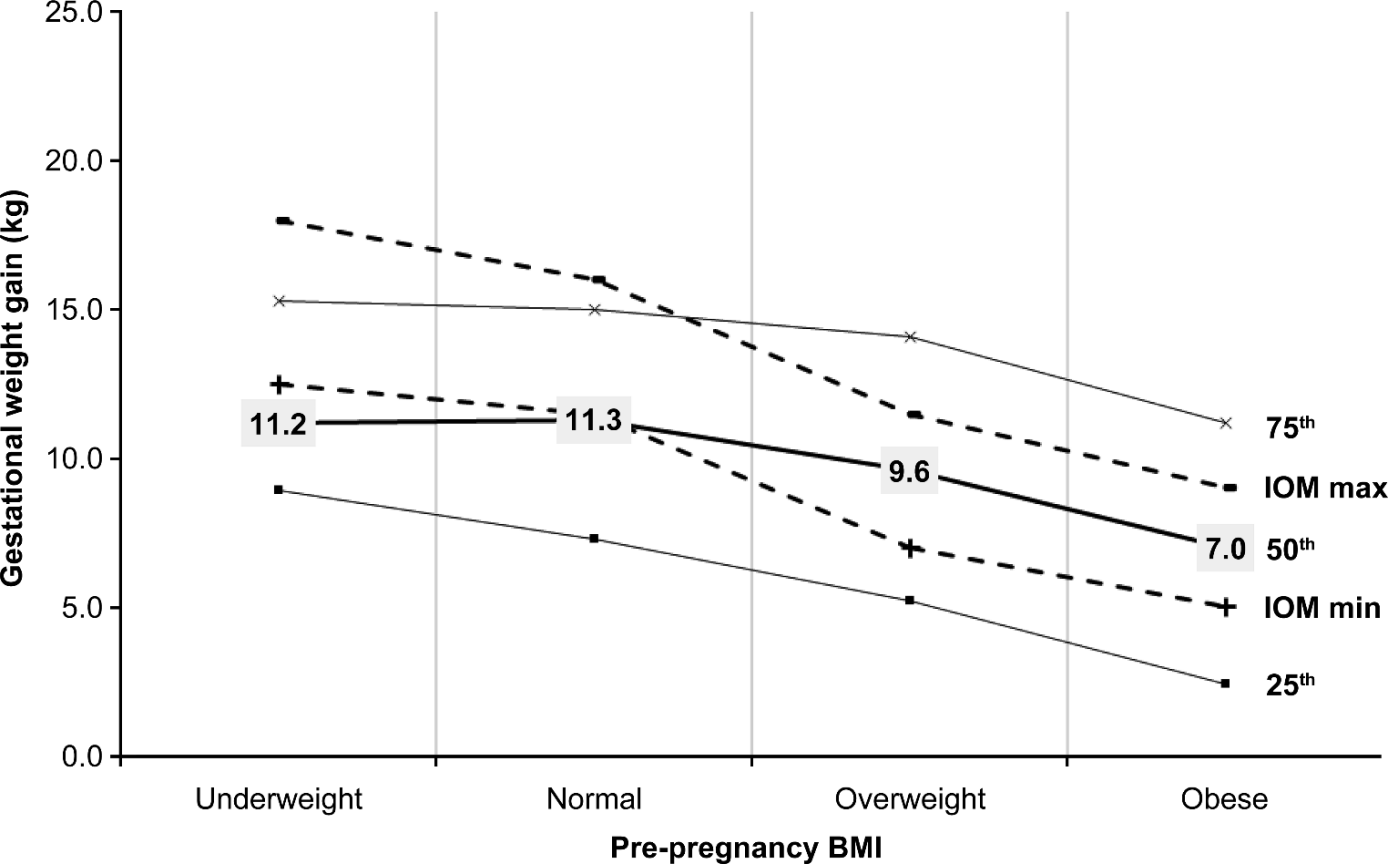
Prepregnancy BMI and gestational weight gain percentiles. IOM ranges (kg): Underweight 12.5–18.0, Normal weight 11.5–16.0, Overweight 7.0–11.5, Obese 5.0–9.0

**Fig. 2:**
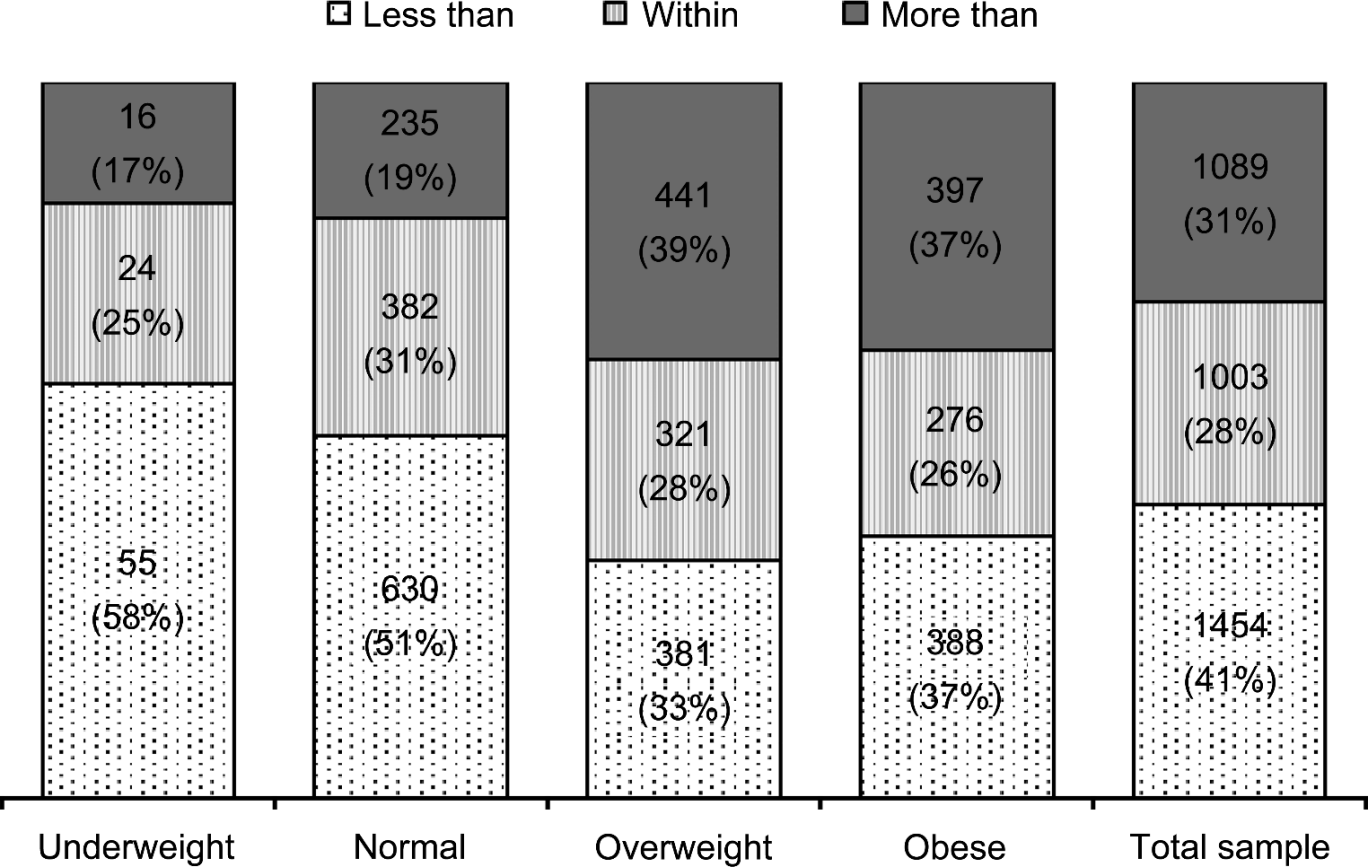
Proportion of women whose gestational weight gain was less than, within, or more than the IOM recommendations, according to body mass index categories

Overall, the mean GWG for the BMI categories were within the IOM’s recommendations (Fig. 1). We found that 41% of all the women women gained less than the IOM’s recommendations, 28% gained within those recommendations, and 31% gained more than those recommendations (Fig. 2). In approximately 83% of women classified as underweight and 82% of women as normal weight, GWG was within or less than IOM values, whereas in 61% of women with overweight and 63% of women living with obesity, GWG was within or less than IOM values (Figs. 1 and 2).

## Discussion

In this study, we examined the relationship between prepregnancy BMI and GWG among women with singleton pregnancies with regard to the 2009 recommendations of the Institute of Medicine (IOM) (Rasmussen & Yaktine, 2009). According to our results, 60% of the pregnant women in our study cohort can either be classified as overweight or living with obesity before pregnancy, and more than 33% each of these groups of women gained excessive weight in pregnancy. These findings corroborate those of other national surveys (Li et al., 2018, 2019; Voerman et al., 2019): that high BMI is prevalent among women of reproductive age and remains a national health burden in Qatar. In this study prepregnancy BMI inversely correlated with GWG, among women with singleton pregnancies. Similar to IOM recommendations, women living with overweight and obesity generally gained less weight proportionally than did women classified as having underweight and normal weight. Similar findings were reported by Daemers et al., 2013; de Jersey et al., 2012 and Johansson et al., 2016. In addition, we also observed that higher maternal age, previous parity, and the presence of diabetes during pregnancy were also associated with decreased GWG. We suggest that this relationship may be partly explained by the previous contact that older, multiparous and diabetic women have had with the health system, which may have increased their awareness about weight and healthy lifestyles. In addition, older and multiparous pregnant women were more likely to have better awareness about achieving appropriate weight gain as a result of experiences with weight retention after previous pregnancies. Cheney et al. (Cheney et al., 2017), however, did not find an association between age and adequate GWG.

In our study, the overall proportion of women whose GWG exceeded IOM recommendations was 31%, and the prevalence of exceeding IOM recommendations for GWG was higher among women living with overweight and obesity, in comparison with those with normal or underweight. In several other studies, the proportion of women who gained more than the IOM recommendations ranged from 27% to more than 60% (Cheney et al., 2017; Brawarsky et al., 2005; Chasan-Taber et al., 2008; Daemers et al., 2013; de Jersey et al., 2012; Olson & Strawderman, 2003; Ferraro et al., 2012; Althuizen et al., 2009), and living with overweight or obesity was a significant predictor of excessive weight gain. However some studies showed that, GWG was most excessive among the overweight group in comparison to other BMI categories based on IOM recommendations (Chasan-Taber et al., 2008; Daemers et al., 2013; de Jersey et al., 2012; Althuizen et al., 2009; Stuebe et al., 2009; Restall et al., 2014). To explain the prevalence of excessive weight gain in women who are overweight or obese reported in the literature, Kominiarek and Peaceman suggested that it was possible that these groups of women required a more intensive form of health behavior program which was not obtainable in pregnancy given the: physiological adaptations that occur in pregnancy, reduced physical activity and the short interval between pregnancy and delivery for GWG goals to be achieved (Kominiarek & Peaceman, 2017).

### Policy Implications

Pregnant women in Qatar have access to multidisciplinary care and management involving obstetricians, endocrinologists, diabetes educators, and nutritionists during pregnancy and the immediate postpartum period. Nevertheless, the current efforts to reduce rates of high BMI among women of reproductive age in Qatar must be sustained to reduce the associated health burden on these women and their offspring. Women living with overweight and obesity with excessive GWG could be at risk for postpartum weight retention and other fetal and maternal complications associated with excessive GWG and high BMI. Women living with obesity, both pregnant and nonpregnant, have access to health management in Qatar, but those classified as normal weight or overweight may not garner much attention and more likely to gain excessive weight during pregnancy, to retain more weight after delivery, and to become obese in the future.

In health care, pregnancy is an ideal time for clinicians to intervene in health matters relating to dietary habits, physical activity, and weight gain (WHO, 2017). The sustained interaction between pregnant women and the health sector throughout the antenatal and postpartum periods should therefore be an opportunity for enhanced, personalized, and integrated management for all pregnant women. Various tools such as counseling and dietary and lifestyle modifications can help pregnant women achieve adequate weight gain and minimize postpartum weight retention.

### Strengths and Limitations

To our knowledge, this is the first examination of the association between BMI and GWG in Qatar in a large and representative sample. Measurement bias cannot be ruled out with regard to the height and weight measurements obtained from hospital records. In addition, the computation of BMI and GWG through self-reported prepregnancy weights is subject to measurement bias, given that women have been reported to underestimate their weights (Kominiarek & Peaceman, 2017). Although it has been shown that the difference is small and it can accurately represent BMI (Brunner Huber, 2007) and is sometimes the only feasible option available (Kominiarek & Peaceman, 2017). Another limitaion is that we did not disaggregate our data based on the various ethnicities of our study population despite questions in the literature on the applicability of BMI and IOM GWG cut-offs which had been derived from Western populations on non-Western populations.

Although the generalised linear model used in this analysis offers more flexibility and is easy to use; it is limited by its sensitivity to outliers and low predictive power. Despite all these limitations, hospital records are an invaluable resource for providing insight into health care practices. These hospital records contain information that inform the day-to-day care and management of patients. Our use of these records was therefore realistic, and according to our examination for selection bias, the findings were generalizable the population of women who gave birth to singleton infants within the study period.

## Conclusion for Practice

A higher BMI was found to be significantly correlated with a lower GWG among women with singleton pregnancies in Qatar. Compared to women classifed as underweight or normal weight, more than one-third of women classified as overweight or obese had GWG that exceeded maximum recommendations. More efforts are needed to study and optimize the GWG pattern in the population of pregnant women in Qatar.

### Electronic Supplementary Material

Below is the link to the electronic supplementary material.

*Online Resource 1*. Method used for computing gestational weight gain and prepregnancy body mass index (BMI). ANC, antenatal care; GA, gestational age.

## Data Availability

Due to institutional regulations, restrictions apply to the availability of data that support the findings of this study.

## Abbreviations

IOM: Institute of Medicine
GWG: Gestational weight gain
BMI: Body mass index
GDM: Gestational diabetes mellitus
DM: Diabetes mellitus
