# Supplementary material for: Maternal Body Mass Index and Recommended Gestational Weight Gain in a Middle Eastern Setting
Source: Matern Child Health J. 2023 Nov 13;28(3):524–31. doi: 10.1007/s10995-023-03816-z (PMC10914897; doi:10.1007/s10995-023-03816-z)
Supplement: Supplementary file 1 — Online Resource 1. Method used for computing gestational weight gain and prepregnancy body mass index (BMI). ANC, antenatal care; GA, gestational age. [file 10995_2023_3816_MOESM1_ESM.docx]

Supplementary figure


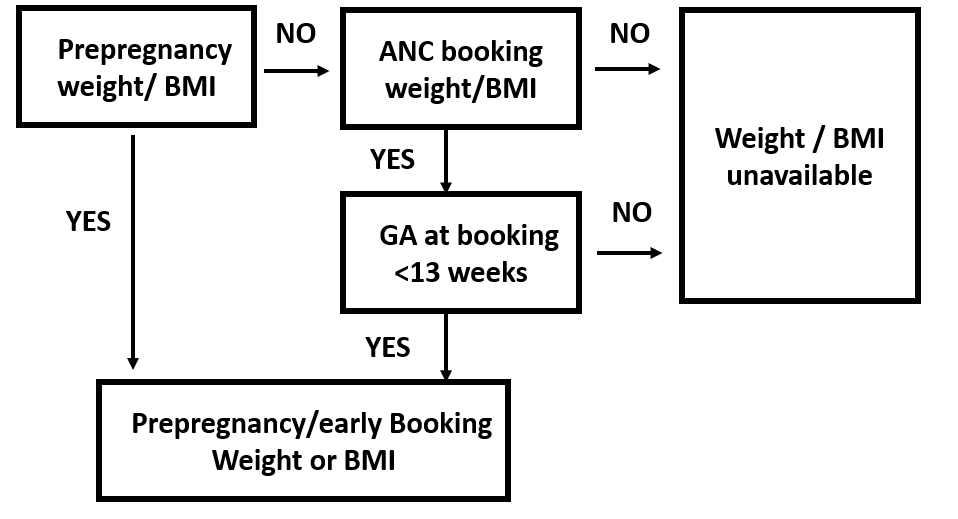


eFigure 1 method used for computing gestational weight gain and pre-pregnancy BMI
